# *In situ* Raman quantitative monitoring of methanogenesis: Culture experiments of a deep-sea cold seep methanogenic archaeon

**DOI:** 10.3389/fmicb.2023.1128064

**Published:** 2023-04-06

**Authors:** Ziyu Yin, Rikuan Zheng, Lianfu Li, Shichuan Xi, Zhendong Luan, Chaomin Sun, Xin Zhang

**Affiliations:** ^1^CAS Key Laboratory of Marine Geology and Environment and CAS Key Laboratory of Experimental Marine Biology and Center of Deep Sea Research, Institute of Oceanology, Chinese Academy of Sciences, Qingdao, China; ^2^Laboratory for Marine Geology and Laboratory for Marine Biology and Biotechnology, Pilot Laboratory for Marine Science and Technology, Qingdao, China; ^3^University of Chinese Academy of Sciences, Beijing, China

**Keywords:** Raman spectroscopy, quantitative analysis, *in situ*, CH_4_–N_2_ gas system, deep-sea methanogenic archaea, culture experiment

## Abstract

Gas production from several metabolic pathways is a necessary process that accompanies the growth and central metabolism of some microorganisms. However, accurate and rapid nondestructive detection of gas production is still challenging. To this end, gas chromatography (GC) is primarily used, which requires sampling and sample preparation. Furthermore, GC is expensive and difficult to operate. Several researchers working on microbial gases are looking forward to a new method to accurately capture the gas trends within a closed system in real-time. In this study, we developed a precise quantitative analysis for headspace gas in Hungate tubes using Raman spectroscopy. This method requires only a controlled focus on the gas portion inside Hungate tubes, enabling nondestructive, real-time, continuous monitoring without the need for sampling. The peak area ratio was selected to establish a calibration curve with nine different CH_4_–N_2_ gaseous mixtures and a linear relationship was observed between the peak area ratio of methane to nitrogen and their molar ratios (*A*(CH_4_)/*A*(N_2_) = 6.0739 × *n*(CH_4_)/*n*(N_2_)). The results of *in situ* quantitative analysis using Raman spectroscopy showed good agreement with those of GC in the continuous monitoring of culture experiments of a deep-sea cold seep methanogenic archaeon. This method significantly improves the detection efficiency and shows great potential for *in situ* quantitative gas detection in microbiology. It can be a powerful complementary tool to GC.

## 1. Introduction

The deep sea is extremely rich in microbial resources, and several microbial communities remain undiscovered, especially in the deep-sea extreme environments (Jorgensen and Boetius, 2007). Because microbial communities are believed to be related to the origin of life and the vast majority of microorganisms are not yet known to us, such a wide range of microbial resources has great potential for exploitation and use. In recent years, researchers have isolated a variety of methanogenic archaea from various habitats (Simankova et al., 2001; Lyimo et al., 2009; Zhilina et al., 2013). Methanogenic archaea and methanogens have great phylogenetic and ecological diversity despite their limited range of metabolic diversity (Liu and Whitman, 2008; Yang et al., 2020). Recently, our team also isolated a novel strain of methanogenic archaea from a deep-sea cold seep. Our preliminary studies have suggested that different environmental conditions can greatly affect its metabolic methanogenic processes. Therefore, to more conveniently and quickly investigate whether methane can be produced under different conditions and to identify under which conditions methane production is the most efficient, *in situ* detection methods for methane production should be developed. Furthermore, in microbiology, in addition to methanogenic bacteria, other types of gases produced through several metabolic pathways by the microorganisms, such as hydrogen sulfide (Kalenitchenko et al., 2017), hydrogen (Jiang et al., 2014), and carbon dioxide (La Ferla and Azzaro, 2001), can be detected using a similar method. The identification of gas production by these organisms can then be quickly performed. This will also bring great convenience to various subsequent biological studies and will certainly assist in rapidly exploring the best reaction conditions and monitoring the reaction process.

In conventional anaerobic culture experiments of marine microorganisms, the Hungate tube is a commonly used small anaerobic culture equipment. The Hungate tube is convenient for setting various substrate conditions to investigate the fermentation process, culture conditions, and optimal reaction conditions (Bowles et al., 2011). However, the gas generated in the tube cannot yet be measured nondestructively using *in situ* methods. Instead, it can only be measured using a typical procedure of collecting a sample with a syringe or gas-tight needle and testing it using gas chromatography (GC) (Ahamed and Ahring, 2011). GC is widely used for evaluating the gas composition because of its high sensitivity and small sample requirements. However, when the number of samples is large and the detection frequency is high, sample preparation is cumbersome and time-consuming for GC testing in addition to being expensive (Chen et al., 2003). Furthermore, GC requires sampling prior to determination, which is followed by a multistep gas-transfer procedure (Drozd and Novák, 1979).

Laser Raman spectroscopy is an excellent method for studying gases (Wang et al., 2011; Hanf et al., 2014), fluids (Facq et al., 2014; Li et al., 2018), and mineral components (Ma et al., 2021). In recent years, Raman spectroscopy has been gradually applied in the studies of microbiology because of its unique advantages of being inexpensive, nondestructive, requiring a short time, exhibiting high accuracy, and enabling *in situ* monitoring (Shope et al., 1987; Picard et al., 2007; Wu et al., 2015; Schalk et al., 2017; Jehlicka et al., 2019; Shi et al., 2020; Osman et al., 2021; Wang et al., 2022). Furthermore, owing to the ability of Raman spectroscopy to detect rapidly the characteristic peaks of several gas–and liquid-phase substances from a closed system, it can be used for the long-term monitoring of gas production. Quantitative analysis of fermentation gases using Raman spectroscopy was achieved by Numata et al. (2013), and they conducted a detailed study on the ratios of gaseous mixtures. Fang et al. (2018) studied the Raman spectral parameters of H_2_ and CH_4_ gaseous mixtures. They found that the peak area and height ratios between CH_4_ and H_2_ were sensitive to composition (i.e., the molar ratio between CH_4_ and H_2_) but were almost independent of pressure. These two studies provided a reference for our study; we considered simulating the real gas production process by using gaseous mixtures to build a calibration curve, followed by the application of the curve to the actual biological sample testing process. Our objective was to propose a new, simple, and fast method that does not require device interfacing. In this method, the test environment and equipment depend entirely on the common conditions in microbiology experiments. Thus, the use of custom devices can be avoided, which ensures the versatility and simplicity.

In this study, we established a quantitative calibration curve for methane and nitrogen in a binary-mixture system based on Raman spectroscopy. The methane content in the culture experiments of a deep-sea cold seep methanogenic archaeon can be monitored quantitatively in real time. This method has the potential to become a novel method for gas quantification alone or in combination with GC in microbiology, with the advantages of being nondestructive, fast, and inexpensive.

## 2. Materials and methods

### 2.1. Strains and culture conditions

Following the process reported in previous studies (Zheng et al., 2021, 2022), deep-sea sediment samples were collected by *RV KEXUE* from a typical cold seep in the South China Sea. These sediment samples were cultured at 28°C for 2 months in an anaerobic enrichment medium containing (per liter of seawater) the following: yeast extract, 0.1 g; peptone, 0.1 g; methanol, 10 mL; cysteine hydrochloride, 0.6 g; and resazurin, 500 μL 0.1% (w/v; the pH was adjusted to 7.0). The cultures were purified via the repeated use of the Hungate roll-tube method. Single colonies were picked using sterilized bamboo skewers, which were then cultured in the anaerobic enrichment medium. The purity of the isolate was confirmed via repeated partial sequencing of the 16S rRNA gene. Thereafter, a strain of methanogenic archaea (strain ZRKC1), which belongs to the genus *Methanolobus*, was isolated from the deep-sea surficial sediments. Cells of ZRKC1 were motile cocci. This strain grew between 12 and 42°C (optimum 37°C), at pH between 6.5 and 8.2 (optimum pH 7.0) and salinity from 20 to 120 gL^−1^ NaCl (optimum 45 gL^−1^). To study the production of methane from methanol by strain ZRKC1, 100 μL of freshly incubated cells were inoculated in 10 mL of basal medium (including 2.0 g of the yeast extract, 1.0 g of NH_4_Cl, 1.0 g of NaHCO_3_, 1.0 g of CH_3_COONa, 0.5 g of KH_2_PO_4_, 0.2 g of MgSO_4_ 7H_2_O, 0.6 g of cysteine hydrochloride, 500 μL of 0.1% (w/v) resazurin in 1 L filtered seawater, and pH = 7.0) supplemented with 100 μL methanol at 25°C for 12 days.

### 2.2. Raman spectrometer and data processing

In the experiment, a Raman insertion probe (RiP) system was used to collect the Raman spectra of the gas above the liquid medium in the Hungate tubes. The system description has been detailed in the study by Zhang et al. (2017) and a similar concept for this system was first proposed by Brewer et al. (2004). The system primarily consists of a diode-pumped neodymium-doped yttrium aluminum garnet pulsed laser with a power of 150 mW and wavelength of 532 nm (Kaiser Optical Systems, Inc.) and cooled charge-coupled device (CCD) of 2,048 × 512 pixels (Andor Technology, Inc.). The spectral range (100–4,325 cm^−1^) was split into two regions (100–2,100 and 2,100–4,325 cm^−1^) on the surface of the CCD. The acquired spectra were a combination of these two regions. The Raman spectra were collected using HoloGRAMS 4.1 (Kaiser Optical Systems, Inc.) with an exposure time of 6 s and five accumulations, which is an appropriate monitoring mode based on multiple previous experiments. Spectra were collected 3–5 times per tube at the same focus position using Raman non-contact optics (Kaiser Optical Systems, Inc.) in a dark room with the focus adjusted to the gas above the liquid inside the Hungate tubes. We then used GRAMS/AI® 9.1 software (Thermo Fisher Scientific, Inc.) for the baseline calibration of the Raman spectra. The peak position, height, and area were determined using the GRAMS/AI “Peak fitting” and “Integrate” routine.

### 2.3. Sample preparation and experimental procedures

**Figure 1:**
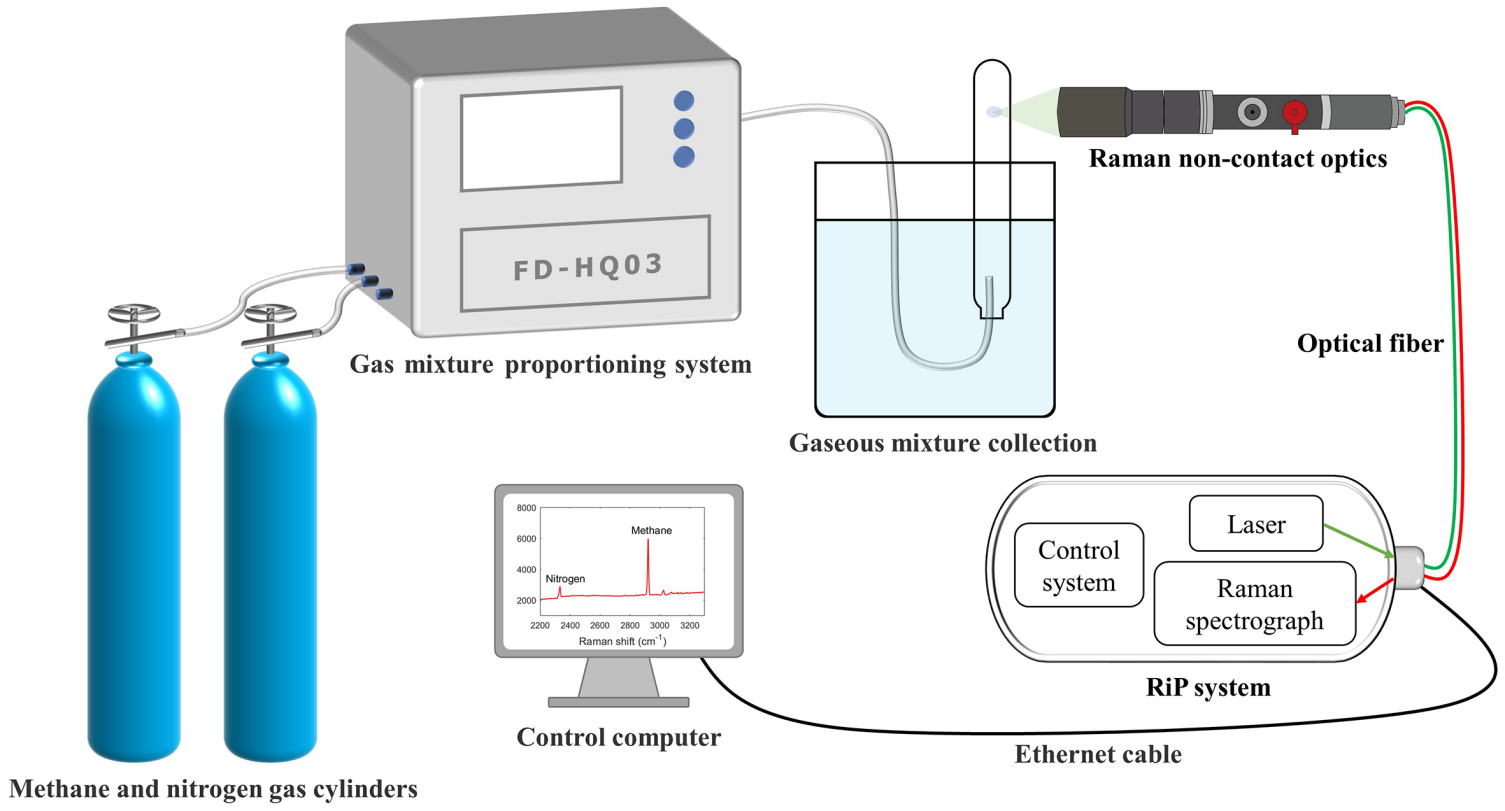
Schematic of the experimental system for establishing the calibration curve. The system is equipped with gas-mixing, RiP, and Raman non-contact optical systems.

In the evaluation procedure of gas component samples with known ratios, we configured nine sets of binary mixtures of methane (>99.9%) and nitrogen (>99.9%) (Qingdao Deyi Gas Co., Ltd.) at different ratios (9:1, 8:2, 7:3, 6:4, 5:5, 4:6, 3:7, 2:8, and 1:9) at a normal temperature and pressure (25°C and 1 atm, respectively) using a multicomponent high-precision dynamic gas distribution system (Suzhou Friend Experimental Equipment Co., Ltd.). Pre-experiments revealed that these ratios adequately covered the dynamics of the gas in the tube during the subsequent methanogenesis of methanogenic archaea. The gas mixture was collected in a large water tank by draining it into the same tube as that used in the subsequent experiments. After stabilization, the Raman spectrum of the gas in the tube was collected by controlling the focus point (Figure 1), and a standard curve of the Raman peak area ratio and gas mixture molar ratio was obtained after processing. Subsequently, additional sets of binary mixtures with different ratios were randomly generated to test the curve accuracy.

**Figure 2:**
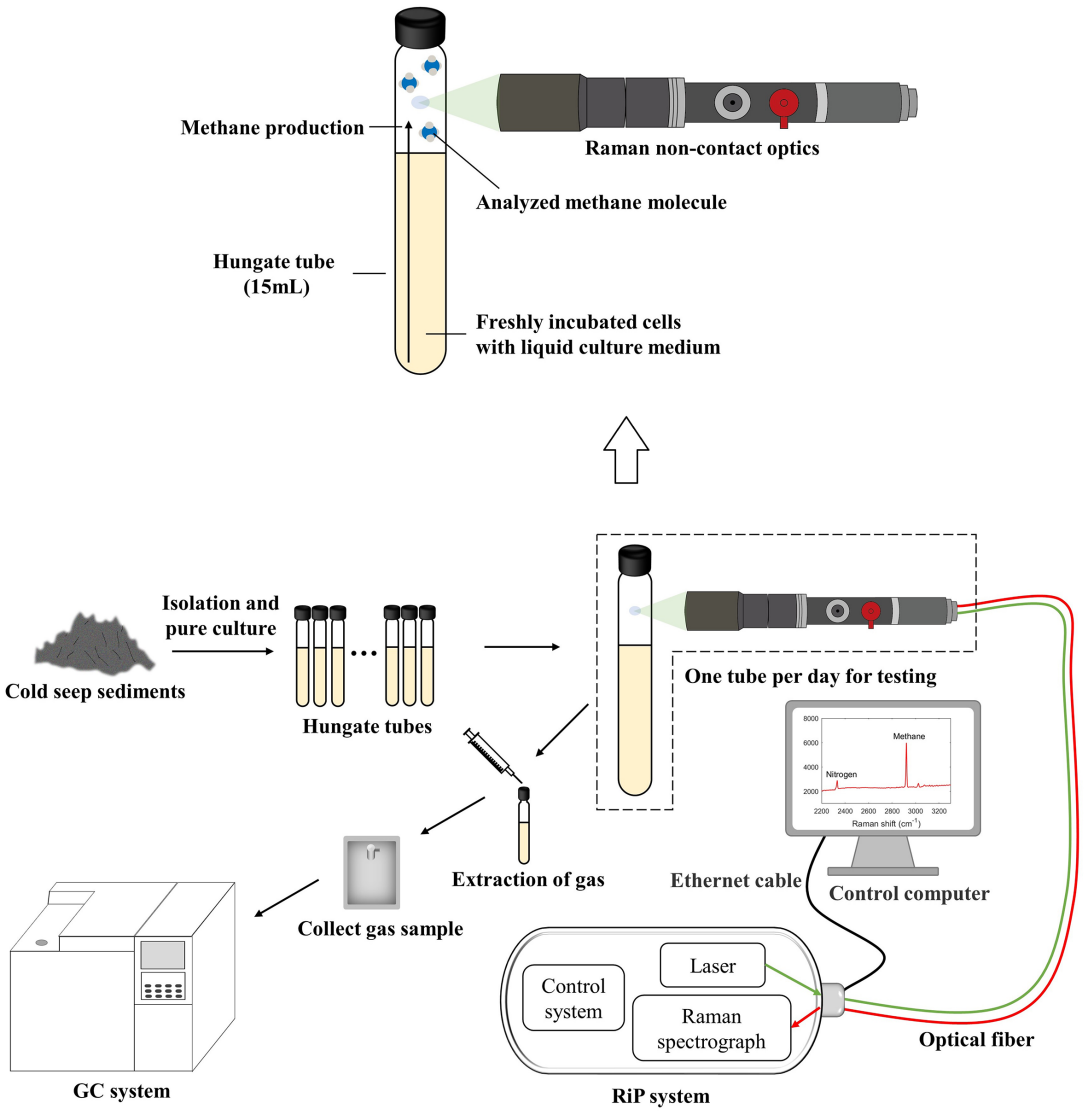
Schematic of the experimental system for the application of the calibration curve to the culture experiments. It shows real-time online *in situ* monitoring of methanogenesis using Raman spectroscopy. The partially enlarged insets illustrate the collection of gas Raman spectra. Raman spectra can easily be acquired by focusing the laser through Raman non-contact optics on the gas portion above the interior of the Hungate tube.

A total of 16 tubes (Hungate tube, 15 ml) with identical initial conditions were configured for the detection of unknown gas production in the actual samples, and a continuous observation period of 10–12 days was planned. The specific operation was to fix the same interval and select one tube for Raman spectroscopy acquisition every day. Subsequently, approximately 5 mL of gas was immediately transferred into a small customized gas bag with a syringe. After this two-step procedure, the sample was not used. After the completion of data collection and sampling, some samples were selected for GC testing as needed (Figure 2), and excess tubes and gas bags were used as spares. In addition to this, for control validation, we selected several tubes for Raman spectra collection at random times in the later stages of the process, and the gas collection was switched to a vacuum blood collection tube directly from the Hungate tube. This method avoids retaining the gas in the syringe and reduces a gas transit step, which may reduce errors and can be used with GC to validate the curve again.

## 3. Results and discussion

### 3.1. Raman spectra of the gaseous mixtures and quantitative theory

**Figure 3:**
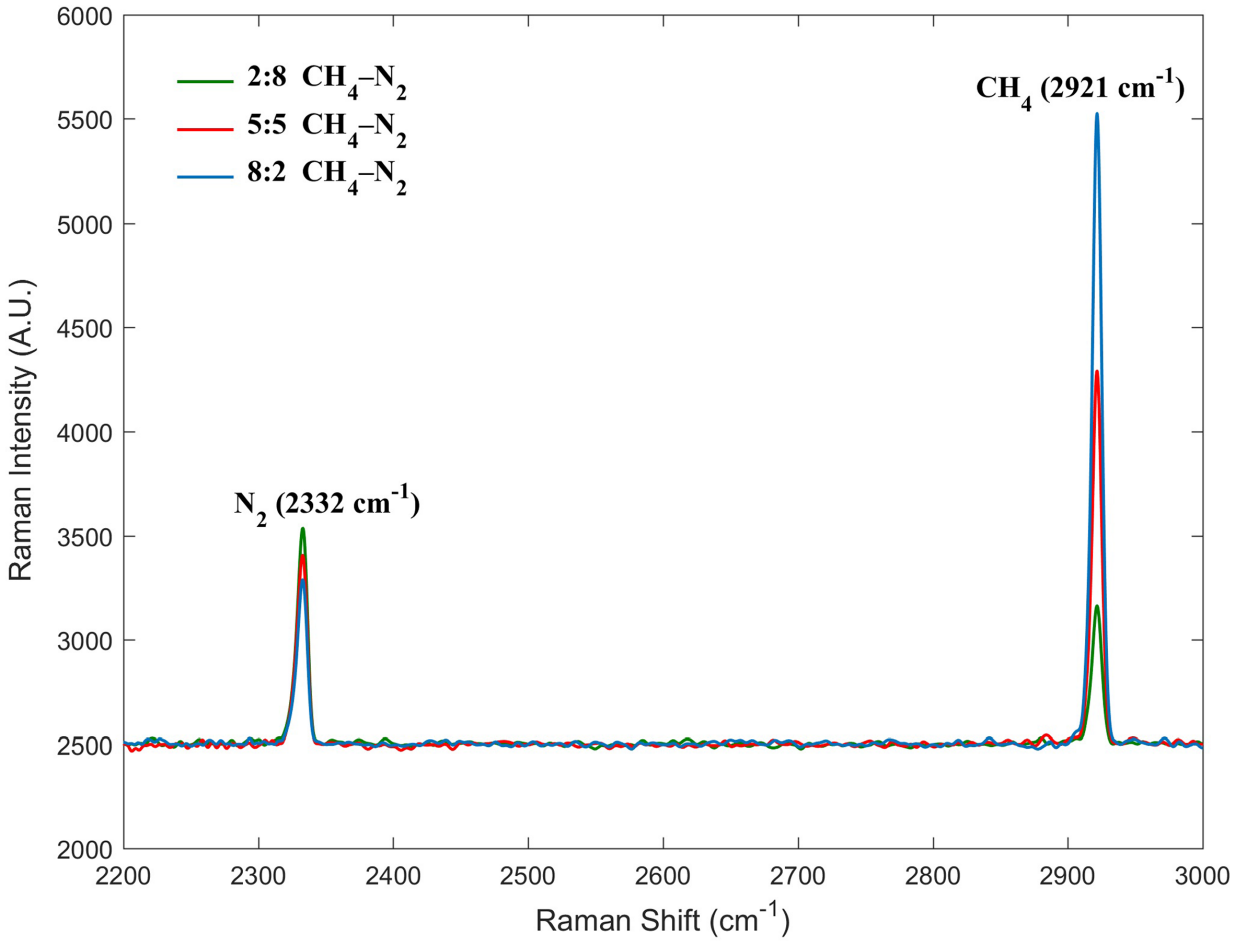
Raman spectra of three molar ratios of methane and nitrogen. The peaks at 2332 and 2,921 cm^−1^ were assigned to nitrogen and methane, respectively.

The C–H symmetric stretching band at 2,921 cm^−1^ was the dominant Raman peak used to identify methane in the CH_4_–N_2_ gas mixture in this study. For nitrogen, the N–N stretching band located at 2,332 cm^−1^ was considered (Figure 3). We uniformly controlled the left and right endpoints of the acquired spectra in the ranges of 2,900–2,940 cm^−1^ for methane and 2,310–2,350 cm^−1^ for nitrogen. All subsequent spectral processing was limited and performed within the respective spectral ranges. The Raman intensity normalization theory of Wopenka and Pasteris laid the foundation for the quantitative analysis of the Raman spectra based on the normalized intensities (intensity ratios) (Wopenka and Pasteris, 1987), which has led to the development of qualitative Raman spectroscopy for quantification. In our experiments, the Raman scattering intensity reflected the amount of gas within the closed system; however, it was also influenced by other factors. The molar ratios of the two Raman-active species, a and b, in a homogeneous phase can be calculated from the peak areas of one of their specific vibrational bands based on the following equation:


$$\frac{A_{a}}{A_{b}}=\frac{C_{a}}{C_{b}}\times \frac{\sigma_{a}}{\sigma_{b}}\times \frac{\eta_{a}}{\eta_{b}}=\frac{C_{a}}{C_{b}}\times \frac{F_{a}}{F_{b}},$$

where *A* is the peak area corresponding to the selected vibration band, *C* is the molar concentration, *σ* is the Raman scattering coefficient, *η* is the instrumental efficiency factor, and *F* is the Raman quantification factor (Wopenka and Pasteris, 1987). This calculation method was widely utilized in the quantitative analysis of gaseous mixtures (Chou et al., 1990; Seitz et al., 1993; Lu et al., 2006; Fang et al., 2018; Chen and Chou, 2022).

To quantify the concentration of methane, known or constant concentrations of Raman-active substances in the sample should be simultaneously measured as a standard reference (Szostak and Mazurek, 2002; Zhang et al., 2016). The Raman peak of nitrogen is a good choice for the procedure of establishing a calibration curve, where it can be used as a quantity of a known concentration. The amount of substance remains constant during the application. Furthermore, nitrogen does not participate in any reactions in this experiment, and it is considered as an inert gas. Consequently, it is well suited to the conditions of the internal standard. In addition, we consistently controlled the environmental conditions. We did not adjust or move the Raman system during the entire experimental cycle and only changed the samples in the sample holder. Therefore, the laser intensity and optical path conditions were consistent. Therefore, the ratio *F*(CH_4_)/*F*(N_2_) can be approximated as a constant. The gas space inside the Hungate tube was always controlled to be maintained at 5 mL. Thus, *C*(CH_4_)/*C*(N_2_) can be calculated from the ratio *A*(CH_4_)/*A*(N_2_), which is also equal to the ratio *n*(CH_4_)/*n*(N_2_).

### 3.2. Establishment and validation of the Raman quantitative curve

**Figure 4:**
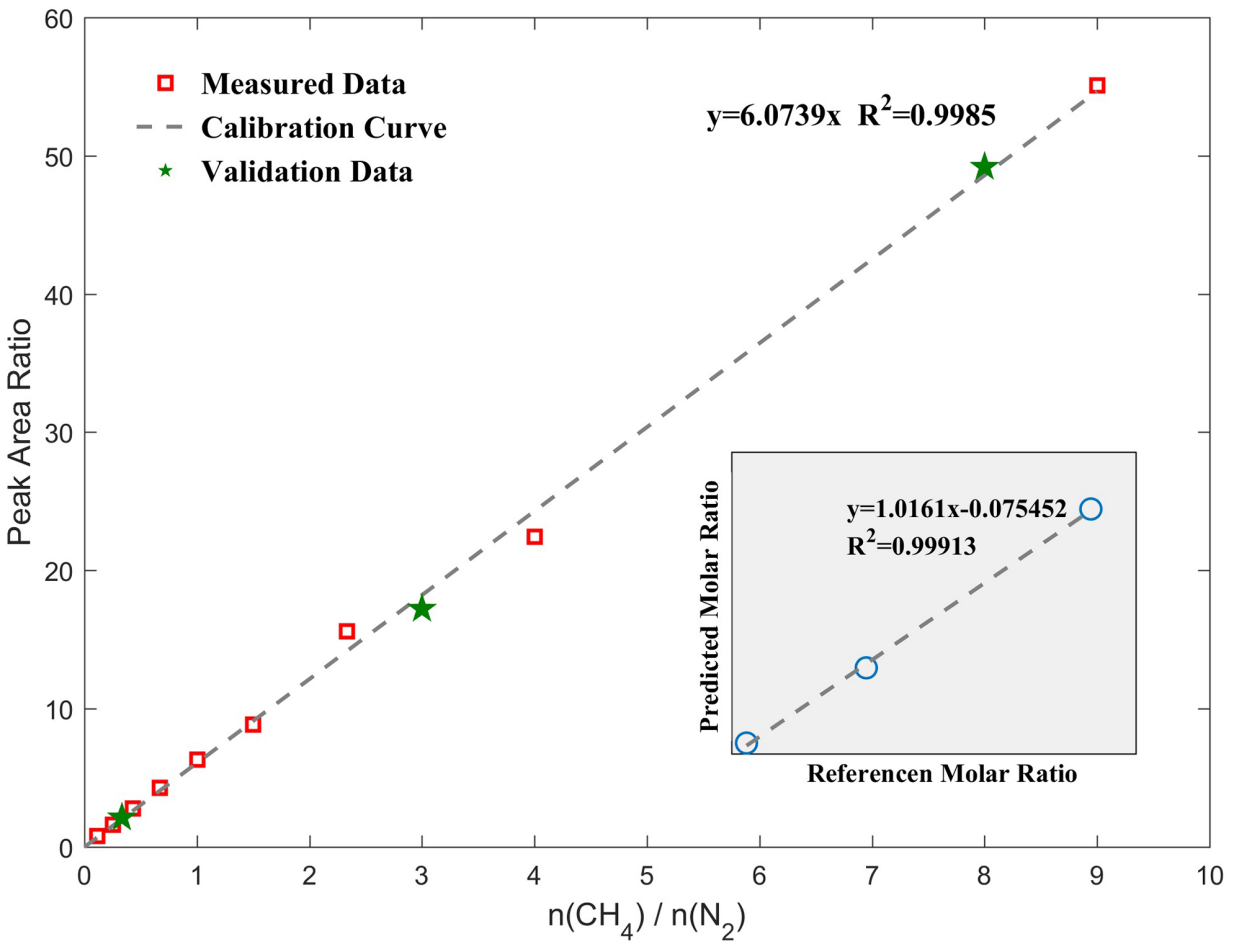
Quantitative calibration curve of the molar ratio of the gaseous mixtures to the Raman peak area ratio. Error bars are within the symbol size; therefore, the relevant values are shown in Table 1. The green stars are validation data that are randomly mixed to test the difference between the theoretical and measured values, and they are close to the calibration curve. The lower-right corner shows the molar ratio predicted by the calibration curve versus the actual value.

Based on our RiP system, Raman spectra of nine different binary mixtures (molar ratios of CH_4_ to N_2_ = 9:1, 8:2, 7:3, 6:4, 5:5, 4:6, 3:7, 2:8, and 1:9) were collected at normal temperature and pressure. We regressed the peak area ratio of methane and normalized nitrogen on the molar ratio of methane to nitrogen to obtain the calibration curve shown in Figure 4. Relevant data are shown in Table 1. In general, different laboratories reported different *F* values due to different Raman systems and optical paths. The slope of the calibration curve is 6.0739 (*R*^2^ = 0.9985), which is representative of *F*(CH_4_)/*F*(N_2_) in this study.

**Table 1:** Molar ratio of methane to nitrogen and the corresponding peak area ratio.

Serial number	Molar ratio	Peak area ratio	Standard deviation
1	1:9	0.780	0.068
2	2:8	1.637	0.074
3	3:7	2.812	0.220
4	4:6	4.230	0.166
5	5:5	6.337	0.350
6	6:4	8.849	0.749
7	7:3	15.630	1.067
8	8:2	22.455	0.062
9	9:1	55.086	1.190

Thereafter, three different ratios of gaseous mixtures were randomly configured, and the volume ratios calculated with their measured peak area ratios were 0.35, 2.84, and 8.10, which were close to the actual values of 0.33, 3.00, and 8.00, respectively. These data are marked with green stars in the standard curve, as shown in Figure 4. The root-mean-square error of the molar ratio based on the calibration curve of the peak area ratio was 0.1095. The slopes of the fitting lines were close to 1.0, which indicates that the curve-predicted and actual values are generally consistent and demonstrates the accuracy of the calibration curve. When only a qualitative detection is required, a signal-to-noise ratio of 3:1 is generally used as the detection limit. In this case, CH_4_ with a volume fraction of 5% in this experiment could be detected by our system. Converting it to a concentration unit is 2.05 mmol/L. A quantitative limit is generally based on a signal-to-noise ratio greater than 10:1. In this study, the signal-to-noise ratios of Raman spectra for both CH_4_ and N_2_ in the detection interval were considerably larger than 10; therefore, the conditions for quantification were available from the spectroscopic point of view. Based on this curve, we could then quantitatively monitor the CH_4_ concentrations in the 10–90% interval in our experiment.

To achieve nondestructive *in situ* real-time monitoring, we used Raman non-contact optics, which have the advantage of a wide range of working distances for remote measurements either directly or through sight glasses and translucent packaging. However, because of its characteristics for remote measurements, other regions through which the laser passes beside the focal point partially excite the scattering effect. This scattering effect will also be reflected in the Raman spectrum, that is, the information in the optical path. Nonetheless, this effect can be eliminated using a simple spectral treatment. Specifically, in this experiment, we used the nitrogen peak area in the Raman spectrum of a Hungate tube filled with pure methane as the reference value of the optical path. The reason for this treatment is that the optical path passes through air containing nitrogen but no methane. This reference value was subtracted from all the peak areas of nitrogen in the subsequent spectral processing to obtain the Raman signal excited only by the nitrogen in the tube under ideal conditions. If we ignore the influence of the optical path, the attempted calibration curve has an *R*^2^ of only 0.8, and nine points appear to have a logarithmic trend, which is inconsistent with the ideal situation. The results showed that the effect of the gas in the optical path cannot be neglected. This data treatment is closer to the actual ideal conditions and more conducive to the establishment of an accurate quantitative curve.

In addition, the Raman spectrum is highly sensitive to pressure and temperature changes (Peercy and Morosin, 1973; Pironon et al., 2003), particularly in a gas (Lallemand et al., 1966; Lu et al., 2007). Therefore, in our experiments, the pressure and temperature were controlled. The characteristics of the methane peaks were systematically investigated by Petrov (2017) in the range of 1–55 bar. The half width of the C–H symmetric stretching band (ν_1_) increased only slightly with increasing pressure (~0.005 cm^−1^/bar). Furthermore, the peak position of the C–H symmetric stretching band shifted toward lower wavenumbers for 1.1 cm^−1^ in the range of 1–55 bar. Two other studies have arrived at similar conclusions (Lin et al., 2007a,b). In our study, the pressure in the culture experiments was estimated to be no more than 5 bar. This was roughly inferred by the insertion of the syringe through the rubber plug into the Hungate tube. Based on Petrov’s model, we showed that both the Raman shift and half-width variations in this range were less than 0.1 cm^−1^. The ν_1_ peak of methane in this range was largely unaffected by pressure; therefore, the effect of pressure on the experiment could be ignored. The study by Lu et al. (2007) contains data that confirm our opinion. Therefore, we did not perform experiments under different pressures in our quantitative analysis. The same concerns applied to the temperature: the culture temperature of the strain was set to normal room temperature (25°C). It is the same temperature as in the previous analysis, and the subsequent continuous observation experiments were also performed at this temperature. Even if there was an error in the controlled temperature, such a small temperature change would not have a significant effect on the experimental results. The claim is also supported by the temperature data reported in a former study (Lu et al., 2007). Therefore, the temperature had no effect on any of the spectra in our experiments.

### 3.3. Application in continuous observation of methanogenesis

**Figure 5:**
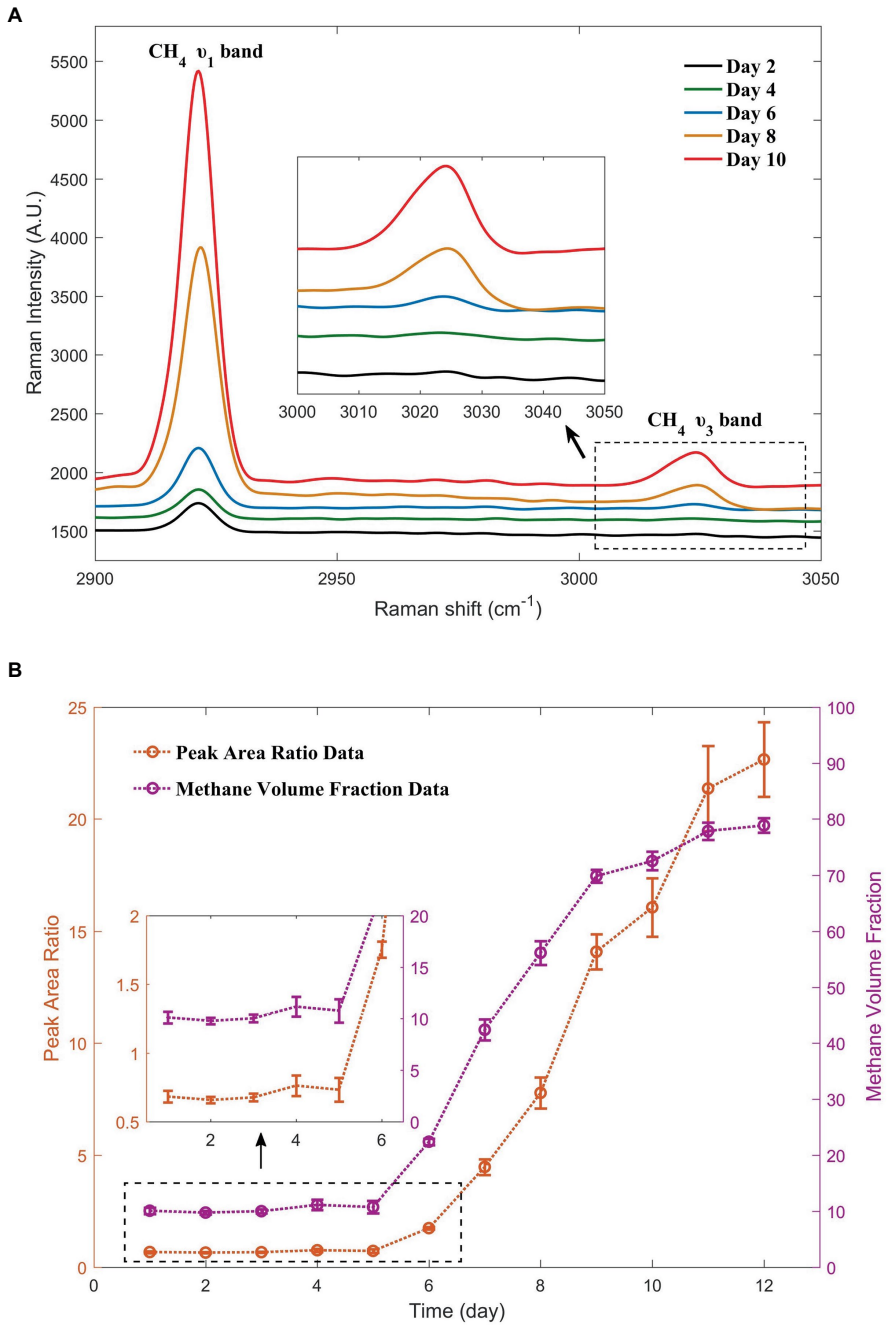
Changes in the methane peaks during the 5 days selected from the 12-day monitoring. The Raman spectra with simultaneous inclusion of nitrogen peaks were shown in Supplementary Figure S1 **(A)**. Methane production, as demonstrated by the methane peak area ratio with nitrogen and methane volume fractions calculated from the curve over a period of 12 days **(B)**.

In the microbiological experiments, the Raman spectra of the gas above the liquid in the Hungate tube were collected over a period of 12 consecutive days. Daily data showed good repeatability. Considering the data of the second day with low methane concentrations in the early stage as an example, the peak area ratio data obtained from five replicates were 0.656, 0.664, 0.697, 0.633, and 0.646, respectively. Figure 5A shows the Raman spectra of the methane peaks collected in the Hungate tube for 5 out of the 12 consecutive days. A clear trend in the intensity of the methane peaks with time were observed, and this trend is presented in two forms in Figure 5B. Based on the trend analysis and the figure, we can surmise that strain ZRKC1 may not have undergone methanogenesis in the first 5 days. At this stage, the observed methane production was low, and the parameters related to the methane peak did not change significantly. From the sixth day, the methane production was noticeable (Supplementary Video S1). In the later stages, methane dominated the gas composition in the tubes. Methanogenesis was suppressed owing to a high production of methane. Furthermore, methanol was almost completely consumed, resulting in the stabilization of the final methane volume fraction. The gas environment inside the tube for the first 5 days was consistent with that of the initial environment, which was a mixed-gas system inside the anaerobic operating table. In the later days, because of a significant methane production, we used the curve to calculate the methane and nitrogen volume fraction data in the tube at various time points. The data are shown in Table 2. The purpose of using volume fractions as units was to enable a better comparison with the GC data.

**Table 2:** Volume fraction of methane and nitrogen calculated from the curve.

Days	Peak area ratio	Nitrogen volume fraction (%)	Methane volume fraction (%)
1	0.684	89.884	10.116
2	0.659	90.210	9.790
3	0.678	89.965	10.035
4	0.764	88.830	11.170
5	0.734	89.225	10.775
6	1.752	77.614	22.386
7	4.470	57.607	42.393
8	7.779	43.845	56.155
9	14.089	30.124	69.876
10	16.070	27.429	72.571
11	21.363	22.138	77.862
12	22.663	21.137	78.863

**Figure 6:**
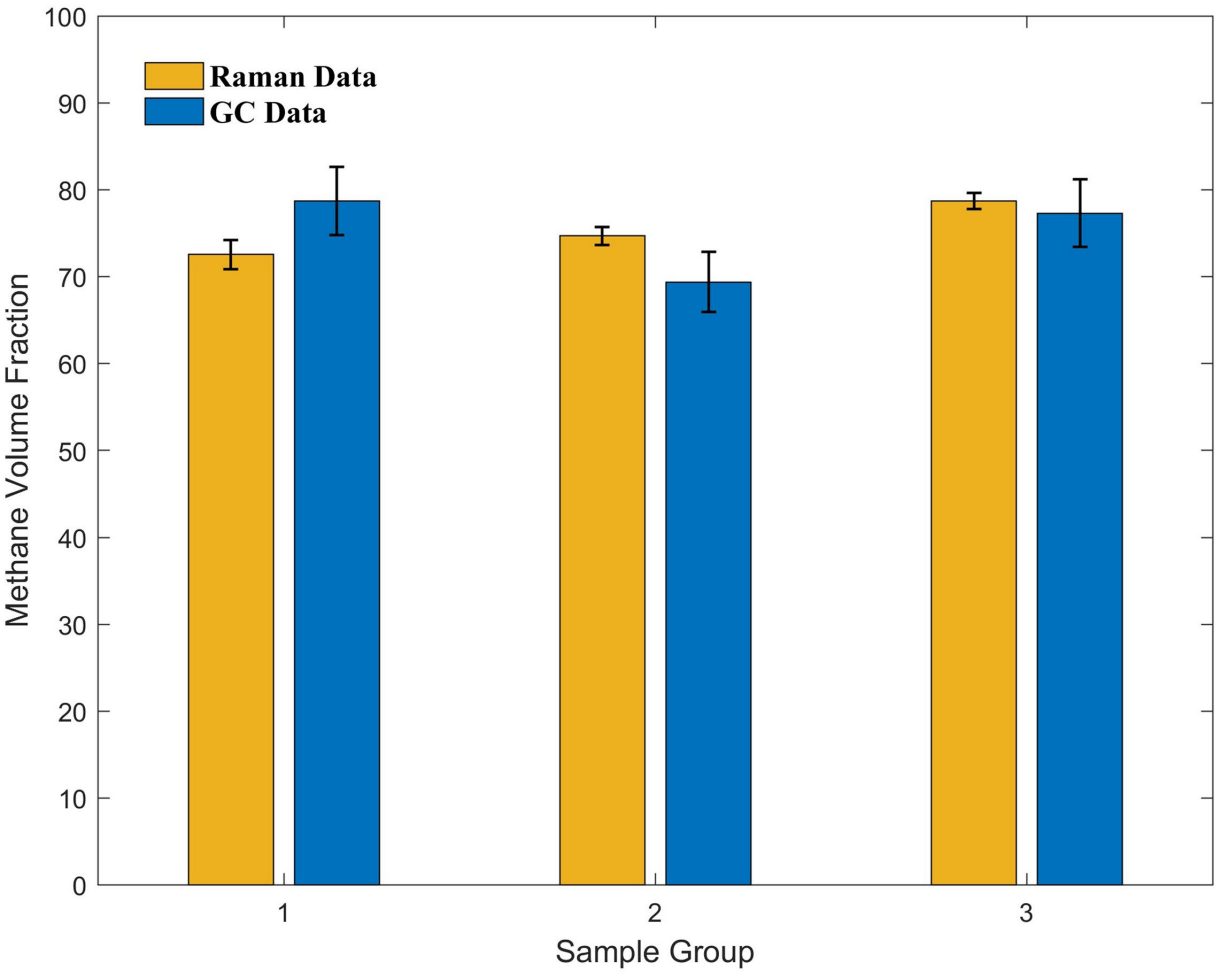
Comparison of the Raman and GC data acquired from three sets of samples with different culture times in the later stages of the culture experiments. The methane volume fraction calculated from the curve using Raman data and methane volume fraction measured using GC show good agreement.

Because the sampling procedure was performed in parallel, we selected several samples for GC testing. Specifically, one sample from day 10 and two samples from the backup group with unknown culture times were selected and sent for testing, yielding methane volume fractions of 78.73, 69.40, and 77.33%, respectively. The peak area ratios for these three data sets were 16.070, 17.931, and 22.357, respectively, and the volume fractions of methane calculated after incorporating them into the curve were 72.57, 74.70, and 78.64%, respectively, which were close to the actual values (Figure 6). A *p*-value of 0.968 obtained from the *t*-test of paired data for the two data sets was considerably greater than 0.05, indicating that there was no significant difference between the two sets of data. Thus, the accuracy of this *in situ* detection method meets the requirements of the observation experiments.

**Figure 7:**
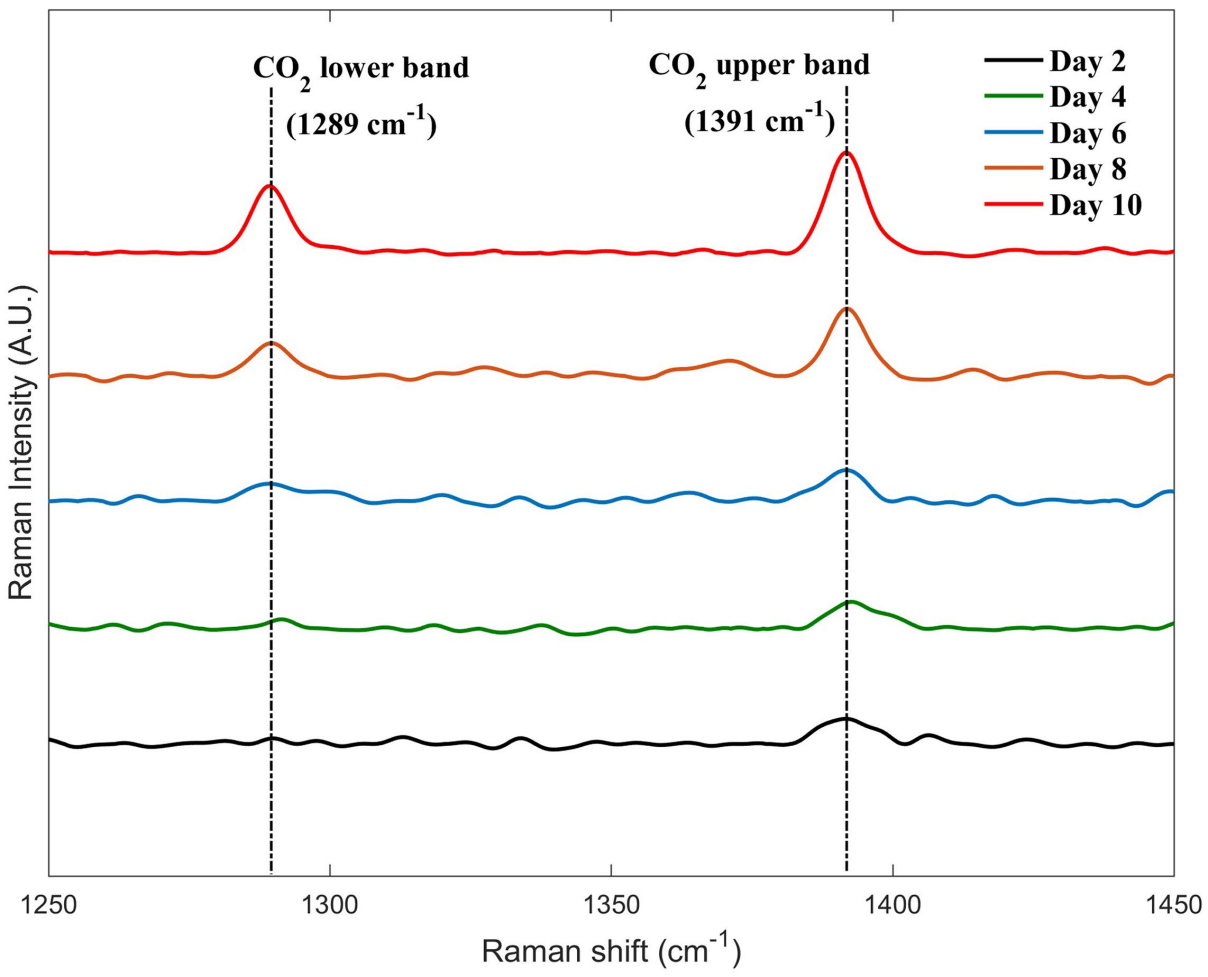
Changes in the carbon dioxide peaks during the 5 days selected from the 12-day monitoring.

Currently, there are five methanogenic pathways for methanogenic archaea, and the corresponding research is abundant. A majority of methanogenic processes are accompanied by the production of carbon dioxide according to the literature (Liu and Whitman, 2008; Zhou et al., 2022). Therefore, when we analyzed the obtained spectra, we focused on the peak of CO_2_, which also showed a slightly increasing trend throughout the 12-day monitoring (5 out of 12-day data are shown in Figure 7). However, we inferred from the qualitative analysis that its production is extremely small. The production of CO_2_ was insignificant compared with the production of CH_4_. Therefore, we neglected the CO_2_ content in the calculated data presented in Table 2. The measured data may not match the theoretical situation in most of the literature, which is an interesting finding. Thus, a trace amount of CO_2_ has a minimum effect on the spectral parameters of methane and nitrogen and can be neglected, which also indicates the need for further metabolic studies on this strain. However, it also shows that a small part of the pressure increase in the tubes during the culture experiments is contributed by the production of CO_2_.

During the continuous observation of methanogenesis, we prepared multiple tubes in the same batch with identical initial conditions for one tube per day of testing. This selection might have incorporated some chance errors, and our data (e.g., the first 5 days provided fluctuating results, and the results on some days were lower than predicted) confirmed the existence of these errors. However, unlike the theoretically ideal method of concentrating on the same tube for daily monitoring with multiple samplings, we discard the tube after collecting the spectrum and taking the sample in the current method. This method does not require multisampling and, therefore, avoids the accompanying problems of signal-to-noise ratio reduction (Numata et al., 2013), pressure reduction (Seitz et al., 1996), and culture contamination. Consequently, this processing allows us to obtain Raman data that are more accurate. We also aim to adopt the former ideal method in subsequent studies that do not require GC validation. Because of the convenience of this method, we can quickly explore whether the strain can produce methane from other organic or inorganic substances apart from methanol, such as shrimp shells and lignin. This method makes it possible to reduce the cost and speed up the assay and facilitates the setting of a wide range of initial conditions, which is ideal for the initial screening of a large number of substances. After the initial screening, further small-scale studies in combination with GC can result in significant time and cost savings.

## 4. Conclusion

In this study, we first established a quantitative Raman curve of methane with nitrogen in a binary mixture system by configuring different mixture ratios. Based on this curve, we quantitatively monitored the metabolism of a novel methanogenic archaeon isolated from a cold seep and successfully demonstrated an *in situ* quantitative detection method for gas production. The curve and its application were separately validated, with fair accuracy. Compared with GC, the proposed method has the following advantages: sampling, gas separation, and transfer are not required and this method enables fast detection and continuous long-term monitoring at fixed time intervals. This will bring great convenience to similar studies by reducing the operational difficulty and threshold. In the future, simulations of gaseous mixtures, including methane and hydrogen, at different temperatures and pressures can be conducted. Combined with Raman immersion probes, this method is expected to be better adapted for *in situ* monitoring of microbial fermentation and metabolic processes in extreme environments.

## Data availability statement

The original contributions presented in the study are included in the article/Supplementary material, further inquiries can be directed to the corresponding author.

## Author contributions

XZ and ZY contributed to the conception and design of the study. Material preparation, data collection, and analysis were performed by ZY, RZ, LL, and SX. The first draft of the manuscript was written by ZY, and all authors commented on previous versions of the manuscript. XZ contributed to the funding acquisition, project administration, supervision, writing, reviewing, and editing. CS and ZL participated in funding acquisition, project administration, and supervision. All authors contributed to the article and approved the submitted version.

## Funding

This research was supported by the following grants: the National Natural Science Foundation of China (92058206 and 41822604), the Strategic Priority Research Program of Chinese Academy of Sciences (XDA22050000 and XDA19060402), Key Project of Ocean Research Center, Chinese Academy of Sciences (COMS2020J03), and the Young Taishan Scholars Program (tsqn201909158).

## Conflict of interest

The authors declare that the research was conducted in the absence of any commercial or financial relationships that could be construed as a potential conflict of interest.

## Publisher’s note

All claims expressed in this article are solely those of the authors and do not necessarily represent those of their affiliated organizations, or those of the publisher, the editors and the reviewers. Any product that may be evaluated in this article, or claim that may be made by its manufacturer, is not guaranteed or endorsed by the publisher.
