# Supplementary material for: In situ Raman quantitative monitoring of methanogenesis: Culture experiments of a deep-sea cold seep methanogenic archaeon
Source: Front Microbiol. 2023 Apr 6;14:1128064. doi: 10.3389/fmicb.2023.1128064 (PMC10115991; doi:10.3389/fmicb.2023.1128064)
Supplement: Supplementary file 1 [file Data_Sheet_1.docx]

Supplementary Material

# Supplementary Figures


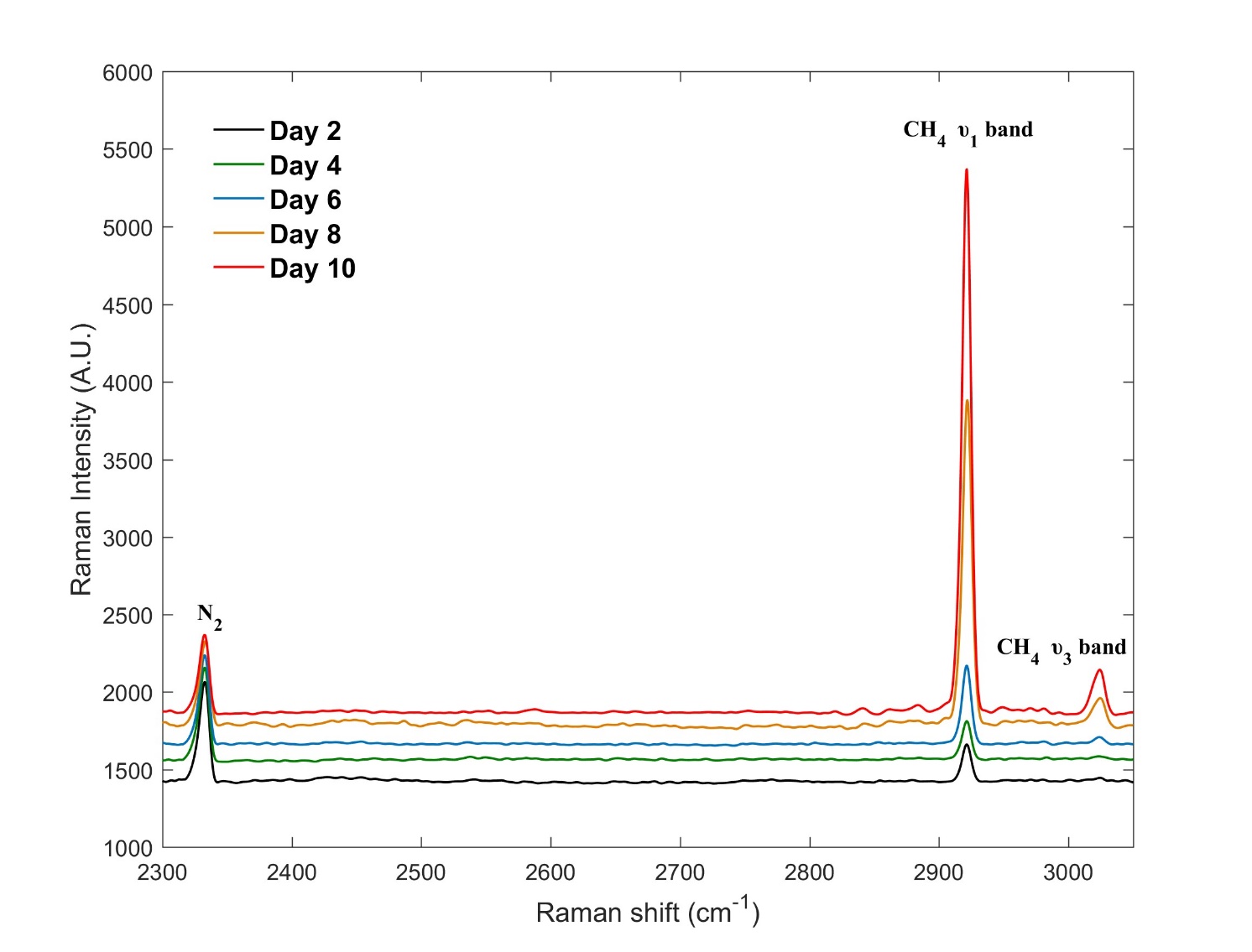


**Supplementary Figure S1.** The supplement to Figure 5 reflects nitrogen simultaneously with methane in a single spectrogram.

# Supplementary Videos

**Supplementary Video S1.** The methanogenesis process is visible to the naked eye of strain ZRKC1.
